# Proposed Meropenem Breakpoints for Interpretation of Antimicrobial Susceptibility Testing of Bacteria Isolated From Dogs

**DOI:** 10.1111/jvp.70067

**Published:** 2026-03-17

**Authors:** Mark G. Papich, Marilyn N. Martinez

**Affiliations:** ^1^ College of Veterinary Medicine North Carolina State University Raleigh North Carolina USA; ^2^ Department of Health and Human Services Center for Veterinary Medicine, US Food and Drug Administration, Office of Generic Animal Drug, Center for Veterinary Medicine, Food and Drug Administration College Park Maryland USA

## Abstract

Meropenem is a carbapenem antibiotic used infrequently in veterinary medicine. However, antimicrobial susceptibility testing standards are needed to monitor for resistance caused by carbapenem resistant Enterobacterales (CRE). The Clinical and Laboratory Standards Institute (CLSI) does not have veterinary‐specific carbapenem breakpoints for testing bacteria isolated from animals. Our objective was to correct this deficiency and propose clinical antimicrobial susceptibility testing breakpoints for consideration by clinical laboratories, standard‐setting organizations, and for monitoring and surveillance programs. We collected pharmacokinetic data from published studies, which were entered into a forecasting program to perform 10,000 Monte Carlo Simulation using a range of dosages considered for administration of meropenem to dogs. Our target for the simulations was a 90% Probability of Target Attainment (PTA) to reach a time‐above‐MIC for the unbound fraction of the drug (*f* T > MIC) for at least 40% of the dosing interval. Our results showed that this target can be met for a MIC of ≤ 1 μg/mL (Enterobacterales) with a dose of 20–30 mg/kg IV every 8 h or 10 mg/kg every 6 h. For a one‐step higher dilution of ≤ 2 μg/mL ( *Pseudomonas aeruginosa* ), a dose of 20–30 mg/kg every 6 h is needed. Therefore, we propose new clinical MIC breakpoints for antimicrobial susceptibility testing these bacteria isolated from dogs of ≤ 1, 2, and ≥ 4 μg/mL for the Susceptible, Intermediate, and Resistant category, respectively, for testing bacteria of the Enterobacterales, and values of ≤ 2, 4, and ≥ 8 μg/mL, respectively, for testing *P. aeruginosa* .

## 1 Introduction

Meropenem is a beta‐lactam antimicrobial agent of the carbapenem group. Like other carbapenems, it was on the World Health Organization Critically Important list (6th revision), but in the newest edition are placed in the “Authorized for Human Use Only” category (WHO 2024). Therefore, the use in animals is discouraged unless it is a *last resort* treatment for dogs and a culture and antimicrobial susceptibility test using the breakpoints presented here are applied. Carbapenems are not allowed to be administered to animals in some countries.

The emergence of carbapenem resistant Enterobacterales (CRE) underscores the need for accurate antimicrobial susceptibility testing breakpoints. The absence of veterinary‐specific breakpoints has hindered antimicrobial stewardship efforts, surveillance, and monitoring programs. Without an antimicrobial susceptibility testing breakpoint, laboratories may not include meropenem on an antimicrobial susceptibility testing panel or, also alarming, falsely interpret the antimicrobial susceptibility test results using either epidemiological cutoff values (ECOFFs) or human breakpoints (Yarbrough et al. 2020).

In a 2024 commentary on the current state of monitoring antimicrobial resistance in veterinary pathogens, Maddock et al. (2024) highlighted the need for testing meropenem susceptibility of veterinary isolates. They pointed out that, “animal species‐specific breakpoints do not exist for carbapenems,” and “The lack of standardization for veterinary testing and/or reporting of carbapenems and lack of public health oversight for veterinary isolates are strong indicators that cases go unnoticed, furthering the potential for zoonotic transmission and global dissemination.”

Despite this need, the European Committee on Antimicrobial Susceptibility Testing (EUCAST) has no testing standards for any carbapenems against isolates from dogs and there are no veterinary breakpoints for testing meropenem listed in CLSI documents for testing isolates from dogs (CLSI 2024). The reluctance of CLSI to approve breakpoints for testing bacteria isolated from animals hinders antimicrobial stewardship efforts, surveillance and monitoring programs. This deficiency motivated us to develop proposed breakpoints for consideration by clinical laboratories, other standard‐setting organizations, and for monitoring and surveillance programs. We chose meropenem rather than imipenem or other carbapenems for this analysis because meropenem is considered by the CLSI, the CDC, and the EUCAST to represent the best balance of sensitivity and specificity when screening for the presence of carbapenemases (Maddock et al. 2024; CDC 2023; EUCAST 2017; CLSI 2023).

Meropenem activity is partially attributed to stability to serine‐based *β*‐lactamases, including those which hydrolyze third‐generation cephalosporins. Meropenem is slightly more active than imipenem against Enterobacterales and *Pseudomonas aeruginosa* . It is less active against *Staphylococcus* spp. and *Enterococcus* spp. (Edwards 1995). The activity against these gram‐positive cocci is not relevant for our study because we do not recommend testing against these bacteria. Methicillin‐resistant *Staphylococcus* spp. are uniformly resistant to meropenem. While infrequently administered to companion animals in the United States—according to practitioner reports and prescribing surveys (Cousto et al. 2020)—it has been the carbapenem of choice for treatment of drug‐resistant infections in animals caused by bacteria of Enterobacterales and *P. aeruginosa* that have not responded to fluoroquinolones and cephalosporins. These infections occur in skin and soft‐tissue, urinary tract, and respiratory tract. Some infections treated with meropenem are likely Enterobacterales with extended‐spectrum beta‐lactamase (ESBL). But the justification is not known because 65.4% of meropenem prescriptions were initiated without or prior to culture results indicating a need for antimicrobial susceptibility testing standards (Cousto et al. 2020).

Although no FDA‐approved veterinary antibiotics are approved for these indications (Papich 2021), carbapenems are legally permitted for use in nonfood producing animals in the United States (AMDUCA 1994). Because it needs to be administered by parenteral injection at least three times daily, and is a labile antibiotic, it is generally limited to inhospital use or, occasionally, to at‐home subcutaneous (SC) injection by a pet owner. The manufacturer's label states that meropenem for injection vials reconstituted with sterile water for injection may be stored for only 13 h at up to 5°C (41°F). Prior work in our laboratory (Cerreta et al. 2017) showed that 90% strength is maintained for 48 h in the refrigerator, but beyond 48 h the strength declines steadily.

There is no data on efficacy in animals from well‐controlled clinical studies. Clinical use is based on observational studies, anecdotal experience and the opinions of clinical experts. Doses have been published in books, websites, and review articles that were derived from extrapolation of the human dose or data provided in a limited number of pharmacokinetic studies.

Fortunately, the prevalence of carbapenem‐resistant bacteria from companion animals has been low in the United States (Sobkowich et al. 2024). These authors tested 477,426 isolates from dogs and cats in the United States. Carbapenem susceptibility among Enterobacterales isolates was 98.86%, with 0.76% classified as intermediate and only 0.38% as resistant. Despite these apparently hopeful outcomes, the authors noted that due to the absence of veterinary‐specific breakpoints, the criteria used for Susceptible, Intermediate, and Resistant were based upon human breakpoints (Sobkowich et al. 2024). Therefore, these results cannot be interpreted to support the relevance of a particular dose or dosing regimen for the treatment of infectious diseases in dogs. Rather, it supported the importance of continued diligence and surveillance to prevent CRE from becoming a public health risk because of transmission from animals to people.

## 2 Materials and Methods

### 2.1 Pharmacokinetic Analysis

We relied primarily on a pharmacokinetic‐pharmacodynamic (PK‐PD) analysis to propose these clinical breakpoints. We followed the CLSI guidelines for developing new breakpoints for antimicrobial agents used in animals (CLSI 2021). To perform the PK‐PD analysis, we searched the literature using “meropenem, pharmacokinetic(s), and dogs (canine)” as our search criteria.

The means and standard deviations (SD) in Table 1 are presented from the identified published pharmacokinetic studies. Included in the table is the terminal elimination half‐life (T½), volume of distribution at steady state (VD_SS_), volume of distribution area (VD_AREA_) (as estimated from the terminal phase), total (free + protein bound) systemic clearance (CL), and protein binding (when available). To determine the overall mean and variability associated with the pharmacokinetics across all studies, values were corrected for the imbalance caused by differences in the number of subjects per study. To obtain an unbiased estimate of averages and variability in the face of study imbalance, the least squares (LS) mean and its associated variability were calculated (Martinez and Bartholomew 2017). To calculate the variability in the LS means, the sums of squares were determined within each study, multiplying the squared SD of that investigation by its corresponding degrees of freedom. This reflected the within‐study sums of squares. Between‐study sums of squares likewise reflected the squared difference between the cross‐study LS mean value and the mean for the investigation. Within each study, this estimate was multiplied by the number of observations. For the total variability, the within‐ and between‐study variability were added, and the sum was then divided by the overall degrees of freedom (defined as total number of observations (*N*) across all studies (Martinez and Bartholomew 2017)).

**TABLE 1:** Pharmacokinetics of meropenem in dogs.

		Route	*n*	Dose, mg/kg	Half‐life, h	Kel, /h	AUC, mg*h/L	VD_SS_, L/kg	VD_AREA_, L/kg	CL, L/kg/hr	Protein Binding, %	References
Mean	1	IV	5	20	0.68	1.02	76.50	0.257	0.2563	0.264	7.5	Sumita et al. (1992)
SD											4.2	
Mean	2	IV	5	24	0.91	0.79	78.80	0.223	0.3855	0.347		Byun et al. (2016)
SD					0.211	0.183	26.3	0.067	0.1158	0.117		
Mean	3	IV	6	20	0.69	1.03	53.29	0.337	0.372	0.3918		Bidgood and Papich (2002)
SD					0.08	0.13	12.04	0.052	0.053	0.0906		
Mean	4	SC	6	20	0.78	0.88	63.42	0.3579	0.301	0.2649		Bidgood and Papich (2002)
SD					0.23	0.23	14.24	0.079	0.0676	0.059		
Mean	5	IV (CRI)	6	3	0.73	0.96		0.3	0.353	0.339	11.87	Bidgood and Papich (2003)
SD					0.07	0.09		0.15	0.1765	0.1656		
Mean	6	IV	4	20	1.1	0.62	59.10	0.337	0.48	0.33	9.58	Ibrahim and Shwaish (2024)
SD					0.8	0.26	3.12	0.028	0.04	0.04	0.24	
Mean	7	IV	6	120	0.783	0.89	520.00	0.254	0.259	0.24		Harrison et al. (1989)
SD					0.083	0.094						
Mean				37.3	0.8	0.9	141.9	0.3	0.3	0.3	9.7	
SD				39.21	0.267	0.286	190.691	0.089	0.132	0.111	2.186	

*Note:* A blank cell in the table indicates that a value was not reported in the published paper.

Abbreviations: AUC, area‐under‐the‐curve; CL, systemic clearance; Kel, elimination rate; *n*, number of animals in the study; VD, volume of distribution.

### 2.2 Probability of Target Attainment

Monte Carlo simulations, (Ambrose 2006; Turnidge and Paterson 2007) were performed to determine the probability of target attainment (PTA) to reach the PK‐PD threshold for a range of Minimal Inhibitory Concentration (MIC) values and principles described for the administration of meropenem to people (Mattoes et al. 2004). For meropenem, PTA was calculated for a 24‐h interval based on administration of a range of doses and dose intervals administered IV. Although we had access to data from only one PK investigation when administered by subcutaneous (SC) injection, we considered it informative to determine the %PTA via this route because it occasionally is a route of administration in dogs.

For β‐lactam antimicrobial drugs, the time of the free drug fraction above the MIC (*f* T>MIC) is the critical parameter for predicting clinical response using PK‐PD analysis. The *f* in the term represents the unbound fraction (protein unbound) used for the analysis. This fraction provided the basis for correcting total drug pharmacokinetic data collected from published studies. The pharmacokinetic values calculated as described above were entered into forecasting software (Crystal Ball; Oracle Software, Denver, CO, version 11.1.3.0.0) and Monte Carlo simulations generated for 10,000 trials. Data entered for forecasting were the values for the formula below, (MIC, T½, VD_AREA_, CL dose intervals, dose, and protein binding). Population variability in protein binding was not included in our assessments because such estimates would negatively bias our predictions (Toutain et al. 2023; Martinez et al. 2025). The overall mean values and variability were entered as described above. For this analysis, percentage of time the unbound drug concentration was above MIC (*f* T > MIC) for a 24‐h interval was calculated in simulations using the following equation:

$$f\%T>\mathrm{MIC}=\mathrm{Ln}\left(\frac{\text{Dose}\times \mathrm{fu}}{\left[{\mathrm{VD}}_{\text{AREA}}\times \mathrm{MIC}\right]}\right)\times \left(\frac{{\mathrm{VD}}_{\text{AREA}}}{\mathrm{CL}}\right)\times \left(\frac{100}{\mathrm{DI}}\right)$$

where Ln is the natural logarithm, fu is the fraction unbound (1−% protein binding), VD is the apparent volume of distribution (area), CL is systemic clearance, and DI is the dose interval.

A target of *f* T > MIC of 40% of the dose interval was used for PTA calculations, consistent with other studies of β‐lactam antimicrobial agents (Abdul‐Aziz et al. 2015; Berry and Kuti 2022; Trang et al. 2017; Leggett et al. 1989, 1990) This value corresponds approximately to a 1‐log decline in bacterial counts in the neutropenic mouse thigh infection and other infection models. The susceptible PK‐PD cutoff value proposed is the MIC value that attained a 90% PTA of simulated animals (Trang et al. 2017; Leggett et al. 1989, 1990; Turnidge and Paterson 2007).

The PTA threshold for a suggested clinical breakpoint was ≥ 90%, indicating that 90% of the simulated pharmacokinetic values were at or above the threshold considered therapeutically effective for that dose‐MIC combination. The 90% value for PTA is the threshold suggested by others (Ambrose 2006; Turnidge and Paterson 2007) and was used in prior analysis of other antimicrobials from our group.

Although, from a physiological perspective, CL and VD_AREA_ can be viewed as independent parameters, we recognize that the noncompartmental estimates derived from the individual studies can be viewed as correlated (Li et al. 2006). Therefore, we estimated the Spearman correlation to describe the relationship between the reported mean CL and VD_AREA_ values from the investigations included in our analysis. The resulting correlation was 0.75. To explore the influence of including this correlation, our Monte Carlo simulations were repeated for the doses and intervals used in our analysis and presented as a comparison in Supporting Information.

### 2.3 Dosages

There are no approved doses for meropenem in dogs. The dose used in clinical practice has been 8, 10, 12 or 20 mg/kg, every 8 or 12 h based on anecdotal and anonymous lists in tables and websites. By comparison, the human dose is 1 or 2 g per person (14–28 mg/kg) every 8 h IV, or 500 mg per person every 6 h IV.

Most doses cited are for IV administration, but the subcutaneous (SC) route is sometimes used in dogs—and therefore included in our analysis. To model a SC injection, we used a study that documented bioavailability from a SC injection in dogs to be approximately 84% (Bidgood and Papich 2002). Although absorption is less than 100% from the SC route, the non‐instantaneous absorption phase results in a duration of exposure that is similar to that after IV administration. Therefore, given the favorable pharmacokinetics, this may be a convenient route when prescribing a time‐dependent antimicrobial agent such as meropenem (Murray et al. 2025).

### 2.4 Microbiology Data

MIC values were used in our analysis to assess where the proposed breakpoint may fall within, or on either end of the wild‐type distribution.

MIC distributions and the epidemiological cutoff values (ECOFFs; also known as the Wild‐Type Cutoff, and ECV) were obtained from a publicly available database (www.EUCAST.org). It is understood that EUCAST MIC distributions are intended solely for the determination of ECOFFs and are not appropriate for describing or interpreting the epidemiology of resistance. We used meropenem MIC distributions for *Escherichia coli* , as a representative of the Enterobacterales, and *P. aeruginosa* in our analysis.

## 3 Results

### 3.1 Pharmacokinetics

Canine meropenem pharmacokinetic data were available from IV (six studies) and SC administration (one study). These are summarized in Table 1 (with references). LS‐Means were calculated to account for the differences in the number of subjects per study (*n*). The corresponding variability estimates included the within‐study and between‐study variability (where the latter was adjusted for study differences in *n*). The results of this analysis are provided in Table 2. These values were used for Monte Carlo simulations presented below.

**TABLE 2:** Overall summary of pharmacokinetic values used in the analysis following LS‐Means calculation.

LS means values for simulations			
	Half‐life (h)	VD(area) (L/kg)	Clearance (L/kg/h)
*N*	38	38	38
Mean	0.796	0.337	0.310
SD	0.198	0.077	0.071
CV%	24.88	22.88	22.85

### 3.2 Monte Carlo Simulations

**FIGURE 1:**
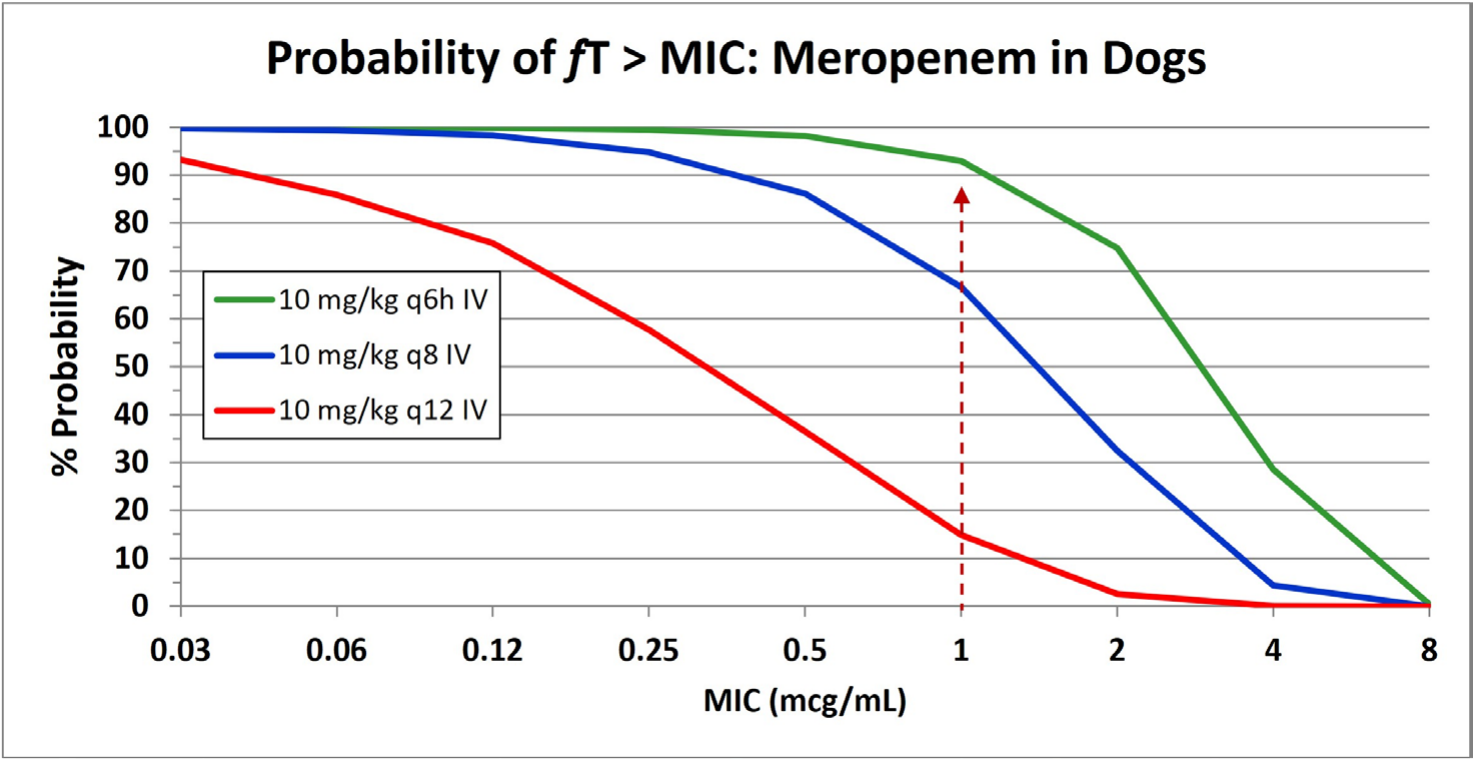
Probability of Target Attainment (PTA) for a range of MIC values for meropenem administered to dogs at 10 mg/kg at 3 dose intervals. The red dashed line corresponds to a PTA of 90% or greater to reach a target of *f* T > MIC > 40% of the dose interval using PK‐PD analysis and Monte Carlo simulations. Note that the 10 mg/kg q8 hrs has a PTA that is less than the targeted 90%. This indicates that at 10 mg/kg, the IV dose would need to be administered q6 hr. to achieve S = 1 μg/mL.

**FIGURE 2:**
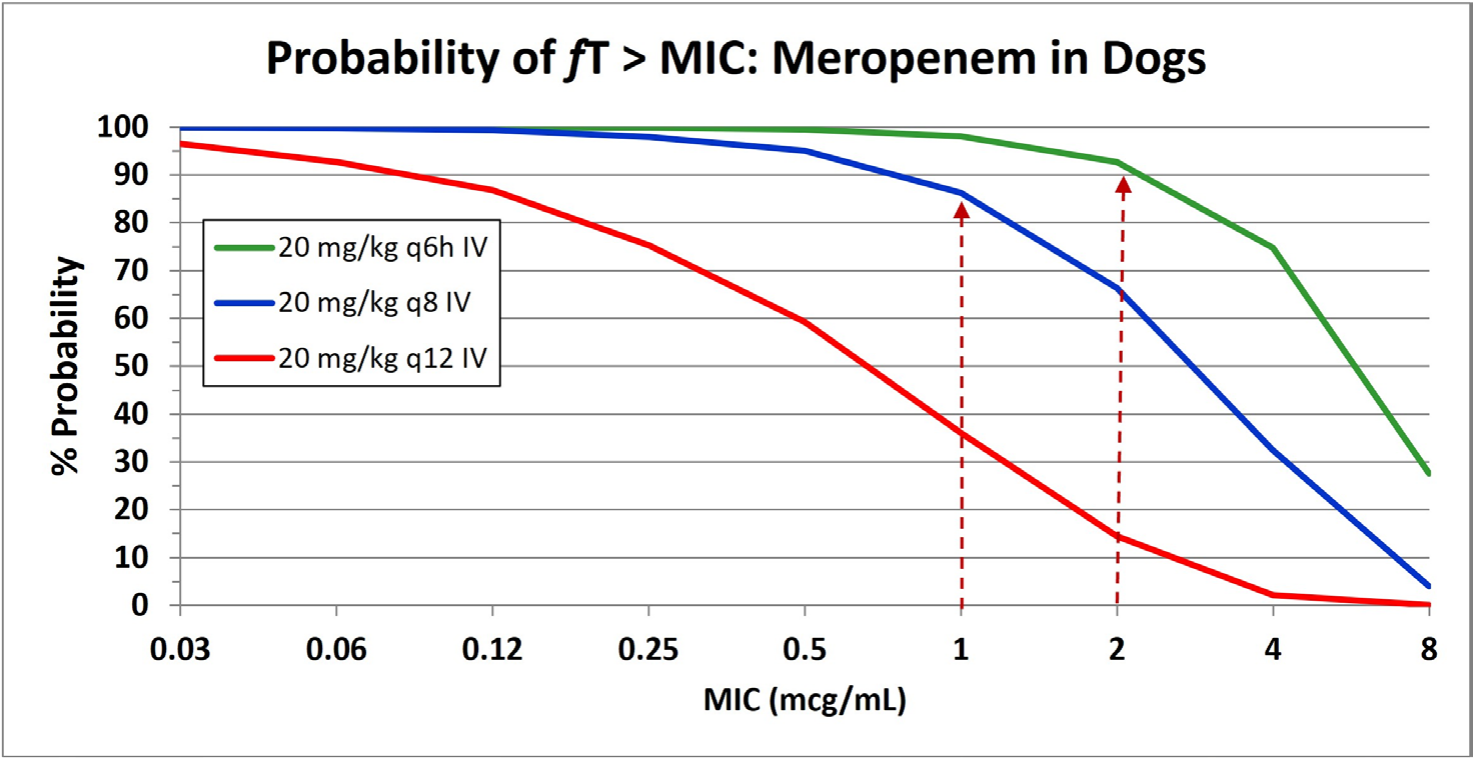
Probability of Target Attainment (PTA) for a range of MIC values for meropenem administered to dogs at 20 mg/kg at 3 dose intervals. The red dashed line corresponds to a PTA of 90% or greater to reach a target of *f* T > MIC > 40% of the dose interval using PK‐PD analysis and Monte Carlo simulations. Note that the 20 mg/kg q8 hrs has a PTA that is slightly less than the targeted 90%. However, we consider a value of 86.25 to be sufficiently close to allow S to be set at 1 μg/mL. As shown by the red dashed line, to achieve S = 2 μg/mL, a dose frequency of q6 hrs is needed.

**FIGURE 3:**
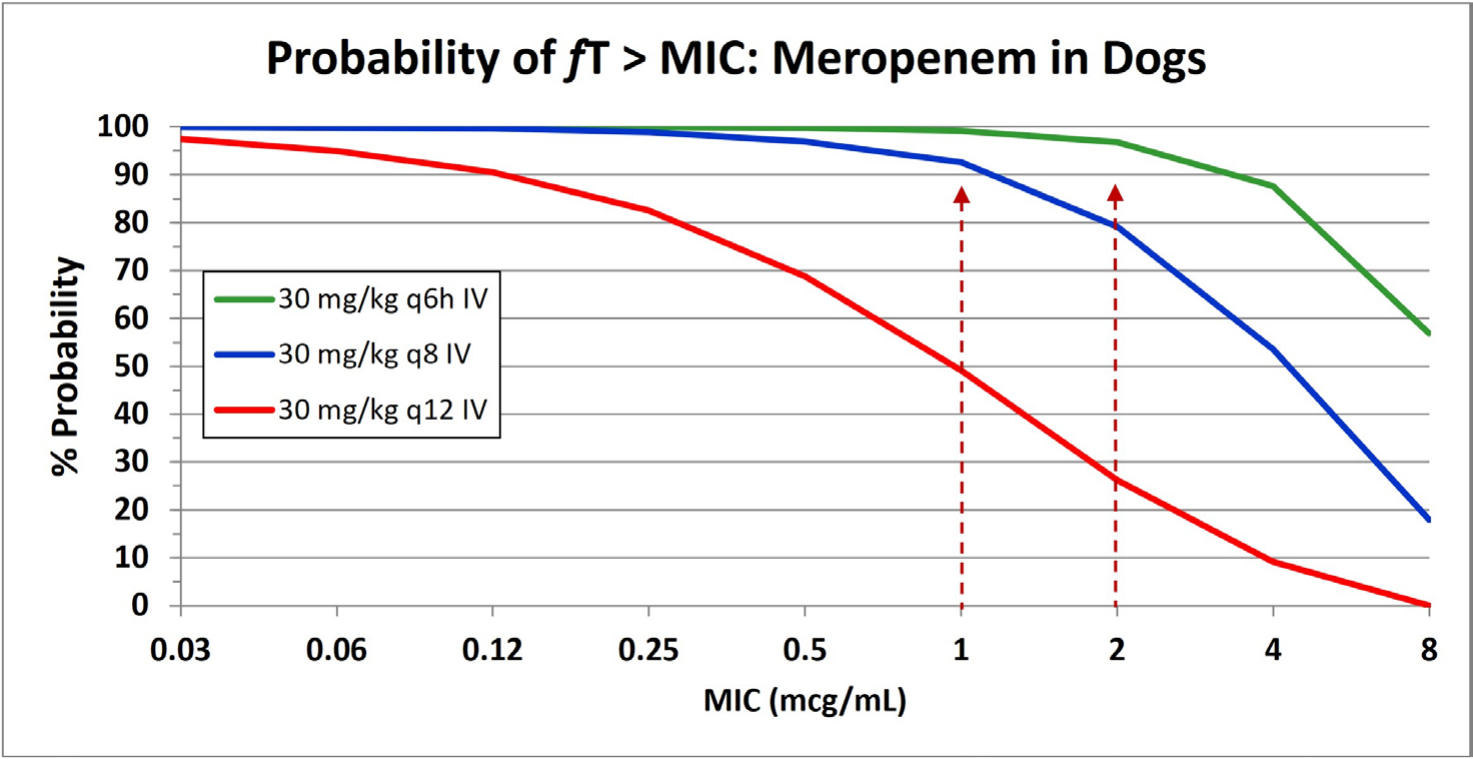
Probability of Target Attainment (PTA) for a range of MIC values for meropenem administered to dogs at 30 mg/kg at 3 dose intervals. The red dashed line corresponds to a PTA of 90% or greater to reach a target of *f* T > MIC > 40% of the dose interval using PK‐PD analysis and Monte Carlo simulations. Note that the 30 mg/kg q8 hrs has a PTA exceeding the targeted 90% at MIC = 1 μg/mL and q6h for S = 2 μg/mL.

The probability of target attainment (PTA) is shown for a dose of 10 mg/kg (Figure 1), 20 mg/kg (Figure 2), and 30 mg/kg (Figure 3), each shown for 3 dose intervals. Because the parameters of VD_AREA_ and CL can be dependent and show correlation, we used a correlation coefficient of 0.75 for these values in additional Monte Carlo simulations and calculation of %PTA. These comparisons are presented in the Supporting Information (Figures S1–S3).

**FIGURE 4:**
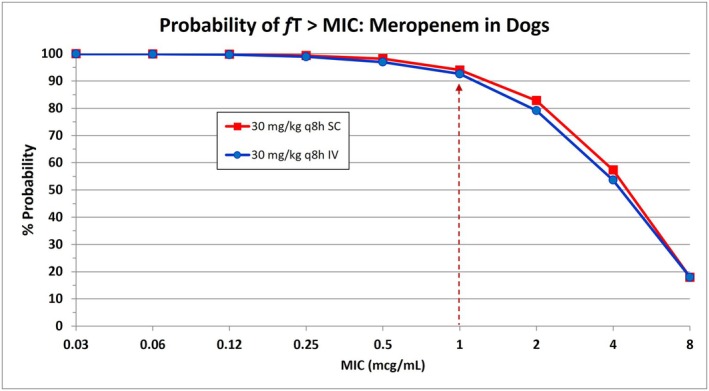
Probability of Target Attainment (PTA) for a range of MIC values for meropenem administered to dogs at 30 mg/kg every 8 h IV versus SC. The red dashed line corresponds to a PTA of 90% or greater to reach a target of *f* T > MIC > 40% of the dose interval using PK‐PD analysis and Monte Carlo simulations. Given that only one set of data were available, estimates of VD_AREA_/CL correlations were not included in this analysis.

We performed a comparison between an IV and SC dose of 30 mg/kg every 8 h because this route is sometimes used in dogs for convenience (Figure 4). Although similar, this analysis shows a slight advantage for the SC administration because of the non‐instantaneous absorption from the injection site.

### 3.3 Microbiology Data

**FIGURE 5:**
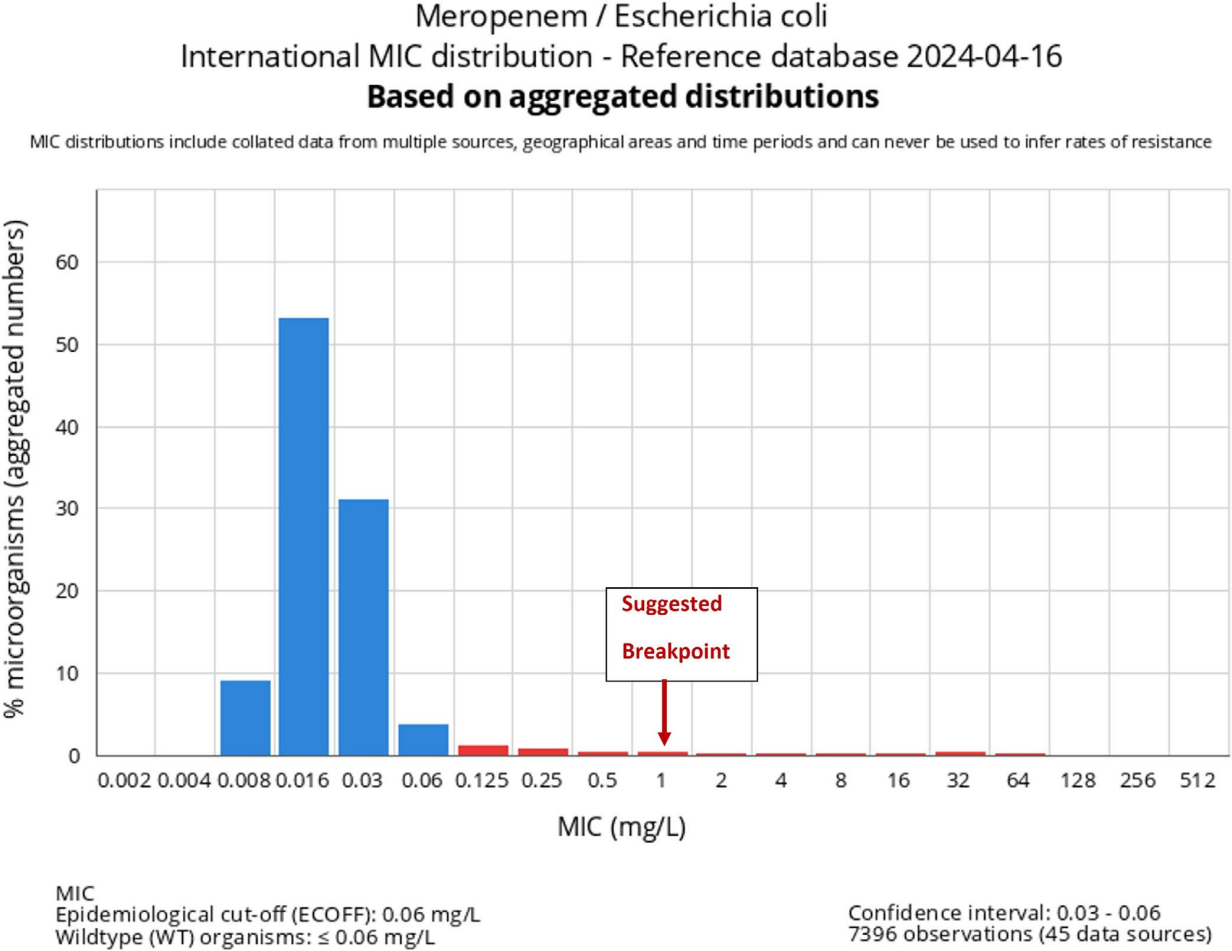
The relationship between the suggested *Escherichia coli* (as a representative of the Enterobacterales) clinical breakpoint for isolates from dogs compared to the MIC distributions. This figure was obtained from EUCAST (www.EUCAST.org).

**FIGURE 6:**
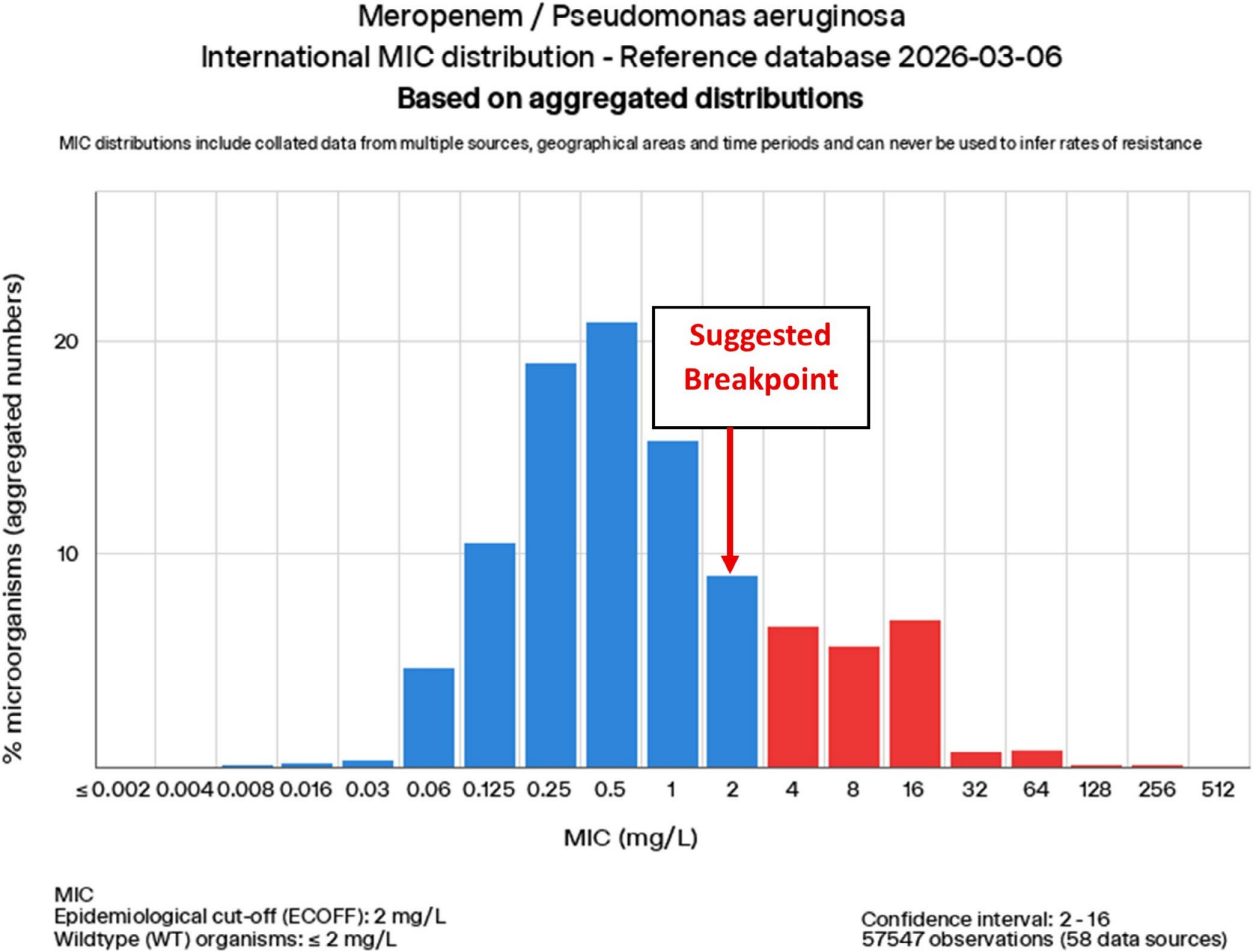
The relationship between the suggested *Pseudomonas aeruginosa* clinical breakpoint for isolates from dogs compared to the MIC distributions. This figure was obtained from EUCAST (www.EUCAST.org).

The MIC distributions and Epidemiological Cutoff Values (ECOFF; also known as wild‐type cutoff values, and ECVs) are shown in Figures 5 and 6. These figures represent the relationship between the proposed clinical breakpoints for Enterobacterales (Figure 5) and *P. aeruginosa* (Figure 6) and the MIC distributions. ECOFF values were accessed from EUCAST, www.EUCAST.org. The ECOFF for *E. coli* (representing Enterobacterales) is 0.06 μg/mL. The ECOFF for *P. aeruginosa* is 2 μg/mL.

### 3.4 Proposed Breakpoint

In accordance with VET02, 4th Ed. (CLSI 2021), when clinical cutoff values are not available (as in the case of meropenem for dogs), the clinical breakpoint can be set on the basis of the PK‐PD cutoff value (CO_PD_). Based on the assessments presented in Figures 1, 2, 3 and assuming an IV dose of 10 mg/kg q6h, or 20–30 mg/kg q8hr (after IV or SC injection), the selected *Enterobacterales* clinical breakpoints are MIC ≤ 1 μg/mL for the Susceptible category, 2 μg/mL for the Intermediate category, and ≥ 4 μg/mL for the Resistant category. We included an analysis using a correlation between parameters, similar to the method by Li et al. (2006). Although including a correlation between VD_AREA_ and CL tended to increase the %PTA for a given MIC, it did not result in a change in our breakpoint predictions, except at a dose of 30 mg/kg q8h (Supporting Information Figure S3). Given that the shift was a function of the reduction in variability attributable to the inclusion of a correlation and given concerns pertaining to potential for the variability to be higher in a patient population, it was prudent to not extend the estimate for “S” when meropenem is administered at a dose of 30 mg/kg. To avoid setting clinical breakpoints in the middle of the wild‐type distribution of *P. aeruginosa* , the proposed clinical breakpoints can be increased by 1 double dilution (≤ 2, 4, and ≥ 8 μg/mL for the Susceptible, Intermediate, and Resistant category, respectively for testing) if doses of 20–30 mg/kg q6hr are employed. Accordingly, practitioners should be advised that when feasible, the meropenem dose should be increased when treating an infection caused by *P. aeruginosa* .

A summary of these values and corresponding doses derived from the analysis in Figures 1, 2, 3 are shown in Table 3.

**TABLE 3:** Suggested Interpretive categories and breakpoint for testing meropenem susceptibility of bacteria isolated from dogs.

	Interpretive categories and MIC breakpoints (μg/mL)			Interpretive category zone diameter breakpoints (nearest whole mm)			Dosage (IV)
	S	I	R	S	I	R	
Enterobacterales	≤ 1	2	≥ 4	≥ 23	20–22	≤ 19	20–30 mg/kg, every 8 h or 10 mg/kg, every 6 h
*Pseudomonas aeruginosa*	≤ 2	4	≥ 8	≥ 19	16–18	≤ 15	20–30 mg/kg, every 6 h

*Note:* The value listed in each cell are the breakpoints (μg/mL) for each interpretive category. The dose corresponding to the S category is listed. The zone diameter refers to the corresponding zone diameter from an agar disk diffusion test performed according to CLSI standards (CLSI 2024). The zone diameters correspond to the same MIC values listed in CLSI M100 (CLSI 2025).

Abbreviations: I, intermediate; R, resistant; S, susceptible.

## 4 Discussion

Without clinical breakpoints for testing susceptibility to meropenem for bacteria isolated from dogs, the inability to classify isolates as susceptible, intermediate, or resistant hinders antimicrobial susceptibility testing laboratories, surveillance and monitoring programs, and epidemiological studies from characterizing the presence of CRE in the canine population. Such bacteria present both therapeutic challenges for dogs with these infections and a public health risk because of a risk of transmission to people, other pets, and the environment.

Our analysis shows that clinical breakpoints for bacteria of the Enterobacterales and *P. aeruginosa* can be attained with dosage regimens shown in Figures 1, 2, 3, and in Table 3. These breakpoints align with the human breakpoints for these bacteria listed in CLSI M100 (CLSI 2025), both in terms of MIC values and zone diameters. Our analysis shows that at the very least, three times daily dosing regimens are needed to reach these therapeutic targets, but in some cases, q6h regimens are necessary. Since meropenem can only be administered by injection, these dosage regimens are often limited to in‐hospital use for serious infections resistant to other antimicrobial agents.

If an IV dose cannot be administered, or difficulty with venous access limits this route, we also examined whether a SC injection would affect exposure. Our pharmacokinetic data for SC administration to dogs was limited to one study (Bidgood and Papich 2002), but showed that similar or slightly higher T > MIC can be achieved from SC administration to dogs (Figure 4). A study in people using SC injection of meropenem also reached the same conclusion of slightly greater exposure (T > MIC) from a SC injection (Murray et al. 2025), which was also well tolerated (as we have found in dogs based on anecdotal experience).

The Enterobacterales breakpoints proposed in this report of ≤ 1 μg/mL (Susceptible) 2 μg/mL (Intermediate), and ≥ 4 μg/mL (Resistant) are somewhat lower than the EUCAST breakpoints and higher than the ECOFF (Figure 5). The rationale for setting the breakpoint higher than the ECOFF is supported by similar decisions for the human breakpoint (Cantón et al. 2014; O'Donnell et al. 2016; Dudley 2012). According to CLSI, when determining if a carbapenem might be a therapeutic option for a patient, it is not necessary to know the resistance mechanism, and reporting results from disk diffusion or MIC tests using current CLSI carbapenem breakpoints is sufficient (CLSI 2017). To reach the 90% PTA in our analysis for Enterobacterales, a dose of 20–30 mg/kg, q8h IV is needed in dogs. However, the ECOFF is 0.06 μg/mL. Because some Enterobacterales isolated from dogs may have MIC values at or below the ECOFF, a lower dose may produce clinical efficacy if an antimicrobial susceptibility test result is available to document a MIC value in this range.

We understand that setting the clinical breakpoint at a value exceeding the ECOFF may raise concerns regarding the potential for treatment failure or the selection of resistant strains. However, these breakpoints are based on the belief that if the systemic exposure is adequate to meet the PK‐PD target for carbapenemase‐producing bacteria, those bacteria can be treated with carbapenems. Our results showed that the MIC associated with 90% PTA in dogs was the same as those classified as “S” for humans. This rationale was also described by Dudley (2012) and Bhavnani et al. (2010). Furthermore, it is likely that many labs will continue to use panels with the human breakpoint. Therefore, it was essential that we vary the dosage regimens to ascertain if the human breakpoints can be achieved in dogs and if yes, at what dose. The proposed breakpoints provide the information to inform practitioners on dosage regimens needed to achieve these values for susceptible strains of *E. coli* and *P. aeruginosa* .

This study has important limitations. (1) The pharmacokinetic data in Table 1 are from studies performed in healthy dogs. We do not know how the pharmacokinetics vary in clinical patients with infection. However, this concern is minimized by the inclusion of uncorrelated PK parameters if one contends that simulations conducted without correlating VD_AREA_ and CL provide an inflated variability estimate. (2) Efficacy of the doses proposed for these breakpoints cannot be confirmed in clinical studies in dogs. There are no reported efficacy studies from the use of meropenem in dogs. Such a study would require a well‐controlled clinical study enrolling many animals and would be impractical.

## 5 Conclusion

Because there are no clinical breakpoints for testing susceptibility to meropenem for bacteria isolated from animals reported by CLSI or EUCAST, we have developed proposed breakpoints that may be considered by laboratories, other standard‐setting organizations, and monitoring and surveillance programs. Based upon the use of a q6h (10 mg/kg) or q8h 20–30 mg/kg, we propose MIC values of ≤ 1, 2, and ≥ 4 μg/mL for the Susceptible, Intermediate, and Resistant category, respectively, for testing bacteria of the Enterobacterales. With a more frequent dosing regimen of q6 hrs for 20–30 mg/kg, we propose values of ≤ 2, 4, and ≥ 8 μg/mL for the Susceptible, Intermediate, and Resistant category, respectively for testing *P. aeruginosa* . These breakpoints align exactly with the human breakpoints listed in CLSI M100, which also provides zone diameters for testing using the agar disk diffusion test. These breakpoints will assist clinical laboratories, surveillance and monitoring programs to detect CRE bacteria that may pose a public health risk.

## Author Contributions

Mark G. Papich was responsible for conceptual planning of the study, data collection, data analysis, and writing the manuscript. Marilyn N. Martinez was responsible for providing advice to the main author on concept and analysis, data and statistical analysis of the data, and writing the manuscript.

## Funding

Mark G. Papich is an employee of North Carolina State University. He received no funding to support the preparation of this manuscript. Marilyn N. Martinez is a Senior Scientist with the United States Food and Drug Administration. She is an unpaid volunteer member of the CLSI, Veterinary Antimicrobial Susceptibility Testing Subcommittee (VAST). She received no compensation for the work on this manuscript.

## Ethics Statement

No animals were used in this study. Therefore, no ethical approval was required as no animals were used. There was no violation of ethical principles during the preparation of this manuscript. The authors adhered to the ethical policies of the journal, as noted on the journal's author guidelines page.

## Conflicts of Interest

Mark G. Papich has no relationship with companies that are sponsors of meropenem. Marilyn N. Martinez is a Senior Scientist with the United States Food and Drug Administration. She has no affiliation with any drug sponsors and is an unpaid volunteer for the CLSI. The views expressed in this paper do not reflect the official position of the FDA. M.G. Papich has been a member of CLSI. M. Martinez is a member of CLSI. However, the analysis and views expressed in this paper do not reflect those of the CLSI‐VAST subcommittee or management. No AI‐assisted technologies were used in the composition of this manuscript.

## Supporting information


**Figure S1:** Probability of Target Attainment (PTA) for a range of MIC values for meropenem administered to dogs at 10 mg/kg at 3 dose intervals.
**Figure S2:** Probability of Target Attainment (PTA) for a range of MIC values for meropenem administered to dogs at 20 mg/kg at 3 dose intervals.
**Figure S3:** Probability of Target Attainment (PTA) for a range of MIC values for meropenem administered to dogs at 30 mg/kg at 3 dose intervals.

## Data Availability

The data that supports the findings of this study are presented in the main body of the manuscript, in Tables and Figures provided, and in Supporting Information provided.
