# Supplementary material for: Proposed Meropenem Breakpoints for Interpretation of Antimicrobial Susceptibility Testing of Bacteria Isolated From Dogs
Source: J Vet Pharmacol Ther. 2026 Mar 17;49(4):367–77. doi: 10.1111/jvp.70067 (PMC13371787; doi:10.1111/jvp.70067)
Supplement: Supplementary file 1 — Figure S1: Probability of Target Attainment (PTA) for a range of MIC values for meropenem administered to dogs at 10 mg/kg at 3 dose intervals. Figure S2: Probability of Target Attainment (PTA) for a range of MIC values for meropenem administered to dogs at 20 mg/kg at 3 dose intervals. Figure S3: Probability of Target Attainment (PTA) for a range of MIC values for meropenem administered to dogs at 30 mg/kg at 3 dose intervals. [file JVP-49-367-s001.docx]

**PROPOSED MEROPENEM BREAKPOINTS FOR INTERPRETATION OF ANTIMICROBIAL SUSCEPTIBILITY TESTING OF BACTERIA ISOLATED FROM DOGS**

Mark G. Papich DVM, MS, ACVCP and Marilyn N. Martinez, PhD

**SUPPLEMENTARY FIGURES FOR REVIEW**

**SUPPLEMENTARY FIGURES**


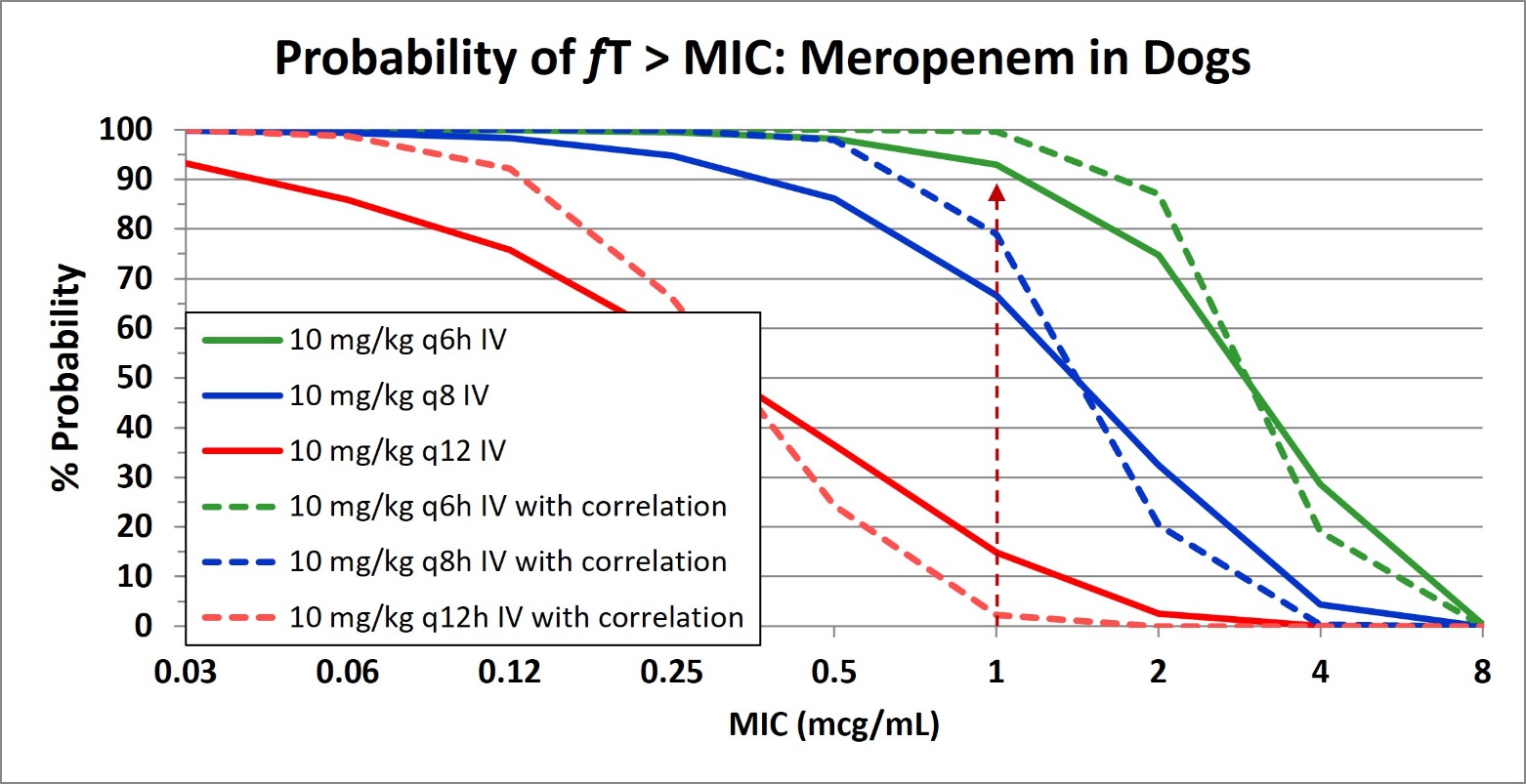


**Figure S-1:** Probability of Target Attainment (PTA) for a range of MIC values for meropenem administered to dogs at 10 mg/kg at 3 dose intervals. The solid lines represent the calculation of the PTA without a correlation between VD_AREA_ and CL. The dashed line of the same color represents the same analysis but with a correlation of 0.75 between VD_AREA_ and CL added. The red dashed vertical line corresponds to a PTA of 90% or greater to reach a target of *f* T>MIC > 40% of the dose interval using PK-PD analysis and Monte Carlo simulations. Note that the 10 mg/kg q8 hrs has a PTA that is less than the targeted 90% with or without the inclusion of the 0.75 correlation between VDarea and CL. This indicates that at 10 mg/kg, the IV dose would need to be administered q6 hr to achieve S = 1 µg/mL.


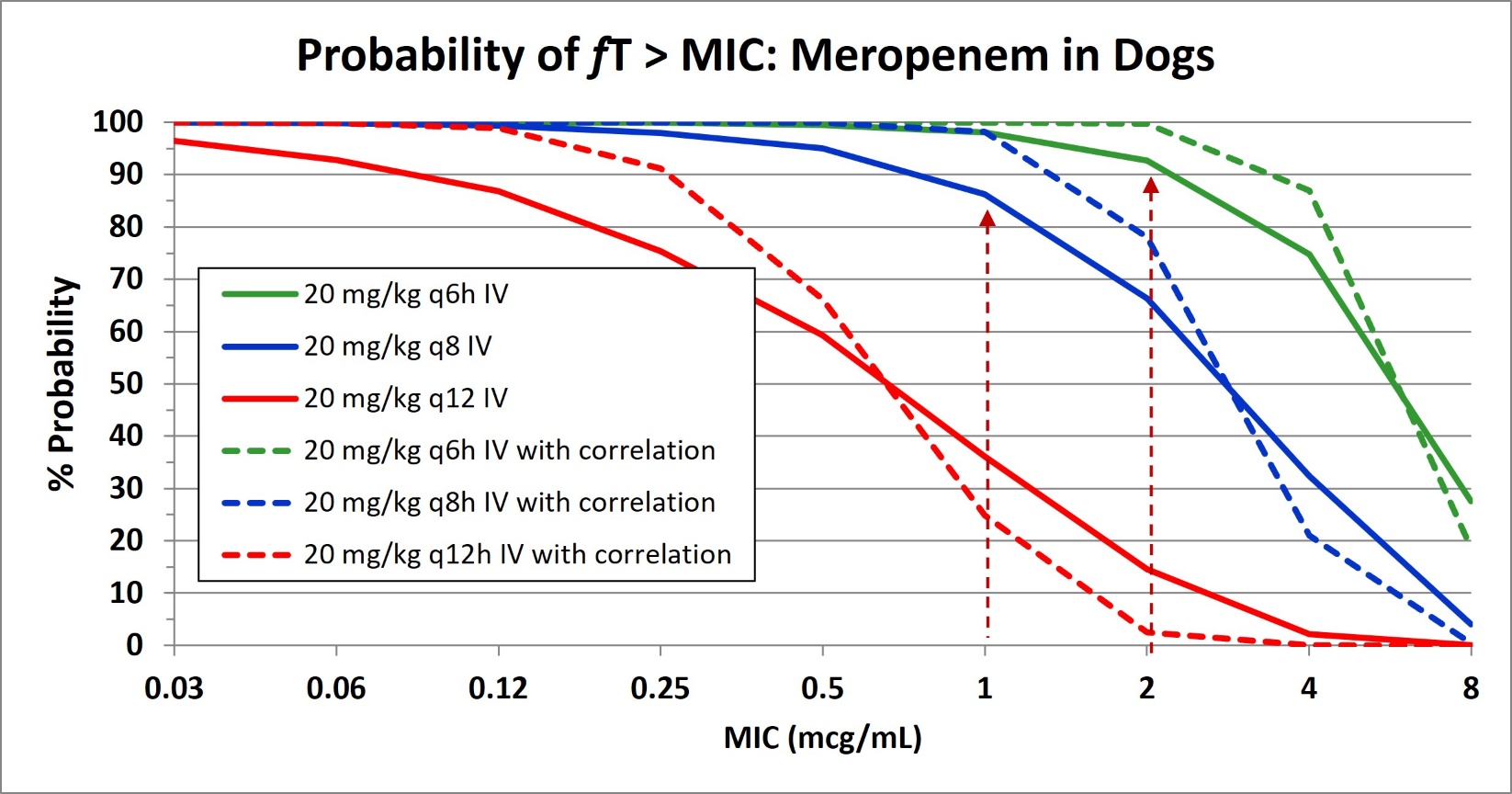


**Figure S-2:** Probability of Target Attainment (PTA) for a range of MIC values for meropenem administered to dogs at 20 mg/kg at 3 dose intervals. The solid lines represent the calculation of the PTA without a correlation between VD_AREA_ and CL. The dashed line of the same color represents the same analysis but with a correlation of 0.75 between VD_AREA_ and CL added. The red dashed vertical line corresponds to a PTA of 90% or greater to reach a target of *f* T>MIC > 40% of the dose interval using PK-PD analysis and Monte Carlo simulations. Note that the 20 mg/kg q8 hrs has a PTA that is slightly less than the targeted 90%. However, we consider a value of 86.25 to be sufficiently close to allow S to be set at 1 µg/mL. To achieve S= 2 µg/mL, a dose frequency of q6 hrs is needed.


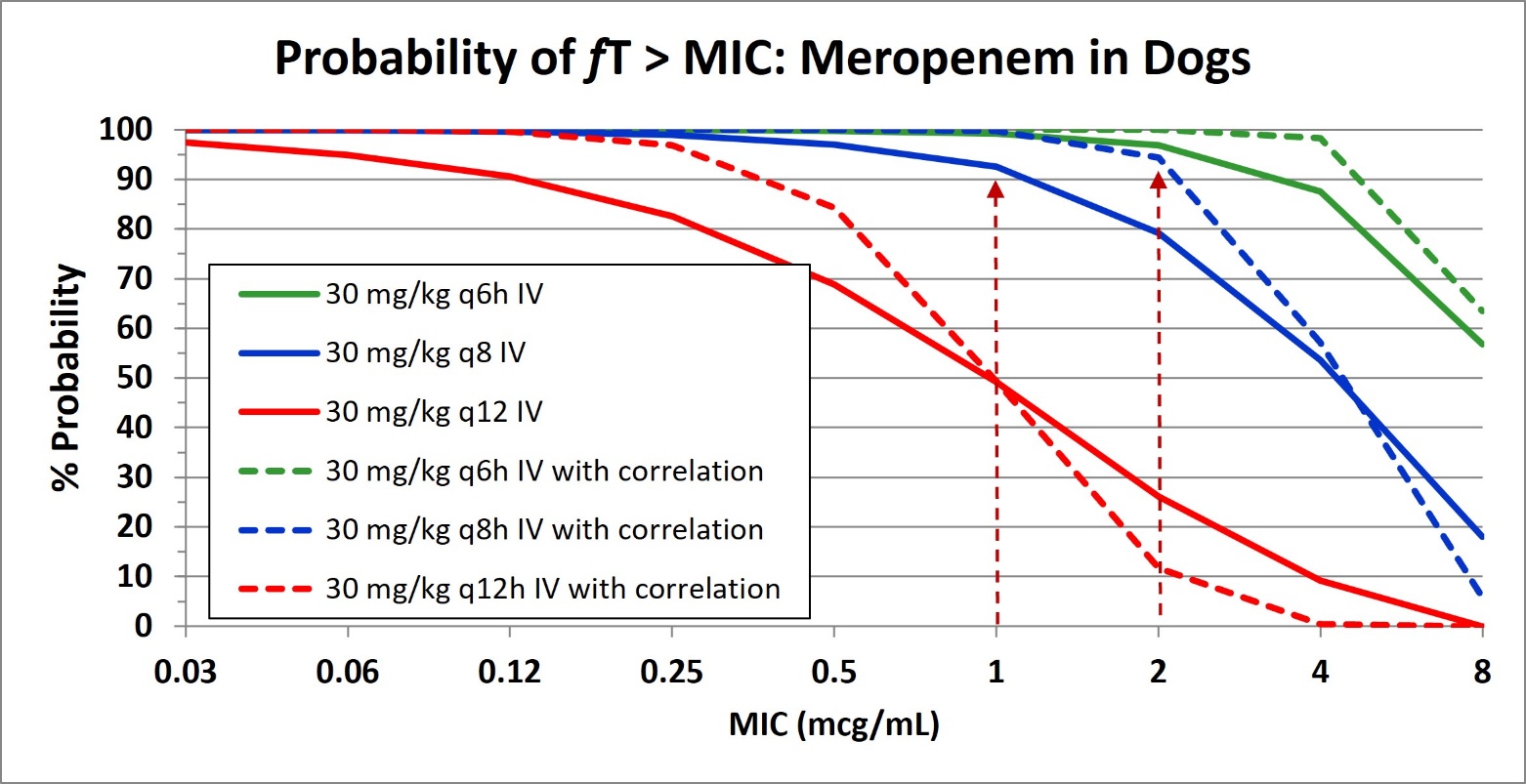


**Figure S-3:** Probability of Target Attainment (PTA) for a range of MIC values for meropenem administered to dogs at 30 mg/kg at 3 dose intervals. The solid lines represent the calculation of the PTA without a correlation between VD_AREA_ and CL. The dashed line of the same color represents the same analysis but with a correlation of 0.75 between VD_AREA_ and CL added. The red dashed vertical line corresponds to a PTA of 90% or greater to reach a target of *f* T>MIC > 40% of the dose interval using PK-PD analysis and Monte Carlo simulations. Note that the 30 mg/kg q8 hrs has a PTA exceeding the targeted 90% at MIC = 1 µg/mL and q6h for S=2 µg/mL. This values is shifted one double dilution for the dose of 30 mg/kg q8h if a correlation of 0.75 is included in the simulations.
